# e-Addictology: An Overview of New Technologies for Assessing and Intervening in Addictive Behaviors

**DOI:** 10.3389/fpsyt.2018.00051

**Published:** 2018-03-01

**Authors:** Florian Ferreri, Alexis Bourla, Stephane Mouchabac, Laurent Karila

**Affiliations:** ^1^Sorbonne Université, UPMC, Department of Adult Psychiatry and Medical Psychology, APHP, Saint-Antoine Hospital, Paris, France; ^2^Université Paris Sud – INSERM U1000, Addiction Research and Treatment Center, APHP, Paul Brousse Hospital, Villejuif, France

**Keywords:** addictive medicine, digital phenotype, ecological momentary assessment, virtual reality, wearable devices, machine learning

## Abstract

**Background:**

New technologies can profoundly change the way we understand psychiatric pathologies and addictive disorders. New concepts are emerging with the development of more accurate means of collecting live data, computerized questionnaires, and the use of passive data. *Digital phenotyping*, a paradigmatic example, refers to the use of computerized measurement tools to *capture* the characteristics of different psychiatric disorders. Similarly, machine learning–a form of artificial intelligence–can improve the classification of patients based on patterns that clinicians have not always considered in the past. Remote or automated interventions (web-based or smartphone-based apps), as well as virtual reality and neurofeedback, are already available or under development.

**Objective:**

These recent changes have the potential to disrupt practices, as well as practitioners’ beliefs, ethics and representations, and may even call into question their professional culture. However, the impact of new technologies on health professionals’ practice in addictive disorder care has yet to be determined. In the present paper, we therefore present an overview of new technology in the field of addiction medicine.

**Method:**

Using the keywords [e-health], [m-health], [computer], [mobile], [smartphone], [wearable], [digital], [machine learning], [ecological momentary assessment], [biofeedback] and [virtual reality], we searched the PubMed database for the most representative articles in the field of assessment and interventions in substance use disorders.

**Results:**

We screened 595 abstracts and analyzed 92 articles, dividing them into seven categories: e-health program and web-based interventions, machine learning, computerized adaptive testing, wearable devices and digital phenotyping, ecological momentary assessment, biofeedback, and virtual reality.

**Conclusion:**

This overview shows that new technologies can improve assessment and interventions in the field of addictive disorders. The precise role of connected devices, artificial intelligence and remote monitoring remains to be defined. If they are to be used effectively, these tools must be explained and adapted to the different profiles of physicians and patients. The involvement of patients, caregivers and other health professionals is essential to their design and assessment.

## Introduction

Addictive disorders are common, but only a small minority of patients receives adequate treatment (1). Diagnosis, early detection of at-risk patients, and the daily monitoring of symptoms and treatments (including self-management) are major issues in addictive medicine and public health. New technologies (smartphone, computers, biomarkers) and the parallel expansion of medical information technology and artificial intelligence have brought about a paradigm shift, resulting in more personalized and predictive medicine (2). These new tools are spurring practitioners to think about addictive disorders in different ways that may ultimately modify their practices. If behavior disorders can be captured by mathematical or computer models, and if diseases can be predicted and relapses detected earlier by machines or smartphones, what role will be left for healthcare teams to play?

These new technologies are also revolutionizing research, insofar as data collection methods can now be classified as either *active* or *passive* (3). The collection of active (or live) data, which refers to all self-evaluation procedures that can be implemented on a computer or smartphone, requires input from the patient, whereas passive data (heart rate, motion detection, sound or light sensor, number of calls sent, duration of calls, etc.) are collected via *background tasks*. Sometimes, patients do not even know when data are being collected, allowing the observer’s influence to be reduced to a minimum.

New technologies also appear to be important in the field of treatment. The expansion of web-based and smartphone-based interventions holds out the prospect of having a coach or therapist in the pocket (4). The democratization of virtual reality and the development of neurofeedback methods also appears useful in addictive disorders.

There are many issues in addictive medicine: pathology screening in patients who sometimes minimize their consumption; active treatment of craving using cognitive behavioral therapy (CBT)-driven techniques applied remotely and in real time; and the strengthening of cooperation between patients and clinicians by facilitating the links between them. The tools currently under development look set to bring many concrete improvements. They may also improve our understanding of the underlying mechanisms of various addictions.

In this article, we conducted an overview of the various innovative technologies related to assessment and intervention in addictive disorders, describing various concepts, applied to addictive disorders, as this new approach could profoundly change the therapeutic relationship, patient assessment and the nature of interventions.

## Method

We conducted a narrative review using the MeSH terms and keywords [e-health], [m-health], [computer], [mobile], [smartphone], [wearable], [digital], [machine learning], [ecological momentary assessment], [biofeedback] and [virtual reality], and we searched the PubMed database for studies in the field of substance use disorders (SUDs). AB, FF, and SM screened 595 abstracts, and 92 articles were then analyzed and divided into seven categories, the first five for evaluation and the last two for treatment: 1. e-health applications and web-based interventions, 2. Machine learning, 3. Computerized adaptive testing (CAT), 4. Wearable devices and digital phenotyping, 5. Ecological momentary assessment (EMA) and ecological momentary intervention (EMI), 6. Biofeedback and neurofeedback, and 7. Virtual reality. These categories were chosen because they correspond to the most innovative topics and most reported in most studies on new technologies in psychiatry.

## Conceptual Overview

A brief description of the concepts underlying new technologies in the field of mental health is summarized in Table 1.

**Table 1:** Summary of concepts.

Concept	Description	Reference
e-health	Combined use of electronic communication and information technology in the health sector. This includes telehealth (health mediated by telecommunications tools: telemedicine, telemonitoring and mobile health/m-health) and robotics (techniques using automatic machines or robots, including machine learning)	(5, 6)
Clinical Decision Support System	Computer-based tool supporting the decision-making process, in order to facilitate organizational processes and provide clinicians with information about patients’ clinical status and the knowledge they need to improve quality of care and patient health	(7)
Machine learning	Scientific discipline that focuses on how computers learn from data, using statistics to learn relationships from data, and computer science to accurately detect classification patterns via efficient computing algorithms	(8)
Computerized adaptive testing, CAT	A computer-administered test in which each item or set of items is selected according to the test taker’s responses to the previous ones	(9)
Ecological momentary assessment	Smartphone-based evaluation of symptoms from day to day in patient’s usual environment, free from recall biases, as the patient self-assesses “*right then, not later; right there, not elsewhere*”	(10, 11)
Ecological momentary intervention	Smartphone-based intervention involving the delivery of psychoeducation, advice or recommendations about how to behave according to the patient’s immediate environment	(12–14)
Digital phenotyping	Moment-by-moment quantification of the individual-level human phenotype using passive data (GPS, accelerometer, voice, call and text logs, screen use) from digital devices (smartphone, wearable devices)	(15)
Biofeedback or neurofeedback	Painless, noninvasive procedure that consists in capturing biometric data (EEG, ECG, EMG, skin conductance, temperature) and feeding them back to patients in real time so that they gradually learn (through positive reinforcement) how to promote brain activity corresponding to the therapeutic target using CBT techniques and relaxation	(16)

## Overview of Technological Innovation Strategies in Substance Use Disorders

### e-Health Applications and Web-Based Interventions

The term *e-health* was originally defined by John Mitchell in 1999 as a “new term needed to describe the combined use of electronic communication and information technology in the health sector. The use in the health sector of digital data—transmitted, stored, and retrieved electronically—for clinical, educational, and administrative purposes, both at the local site and at a distance” (5). This term now covers a broader reality (6), as it includes any device or computer software relating to health, centered around two fields:

1. *Telehealth*, in other words, health mediated by telecommunications tools (telemedicine, telemonitoring and mobile health);
2. *Robotics*, defined as a set of techniques using automatic machines or robots, that includes both medical robotics itself (e.g., robot surgeons) and the use of programs based on artificial intelligence.

It is at the interface of these two fields that new Clinical Decision Support Systems (CDSS) have been developed, defined as computer applications “whose aim is to provide clinicians with information describing the clinical situation of a patient in useful time and place as well as appropriate knowledge … to improve the quality of care and the health of patients” (17). Although e-health technology in the area of SUDs is still at a relatively early stage, several projects are worthy of interest, as these new technologies allow realtime evaluation to be combined with an interventional dimension. Several e-health solutions [i.e., A-CHESS (18)] improve self-management by providing self-assessment modules and reminders and also allow for rapid contact with a support service to ensure swift responses in case of need. Other types of software [ORION (19, 20), D-ARIANNA (21, 22), Steering Clear program (23)] optimize behavioral risk quantification (overdose for Orion, binge-drinking for D-ARIANNA, drink-driving for Steering Clear) via scalar self-assessment modules. These programs deliver rapid intervention in the form of guidelines, tips, motivational techniques and people to contact. Finally some programs [e.g., JITAI (24)] are intended to prevent relapse by providing regular monitoring and individualized coping strategies. A recent review (25) showed that computer-based alcohol interventions are generally effective in reducing alcohol consumption. Longer, multisession interventions are more effective than shorter or single-session interventions. *Other programs* provide individually tailored clinical content in a multimedia format (26, 27) to promote psychoeducation. *In the field of smoking cessation, meta-analysis showed the interest of web-based interventions* (28).

The US Food and Drug Administration (FDA) recently authorized a smartphone-based e-health program for prescription: the Pear^®^ reSET application, which uses CBT, psychoeducation, social connection and self-assessment (craving, mood, etc.) to treat different types of SUDs (except for opioid dependence). The good results (40.3% adherence to abstinence in reSET users vs. 17.6% in control group) nevertheless need to be replicated (data should be available in 2018) (29). A description of these technologies can be found in Table 2.

**Table 2:** Smartphone- and web-based e-health interventions.

Concept	Description
A-CHESS	The Addiction-Comprehensive Health Enhancement Support System includes both static content (e.g., audio-guided relaxation) and interactive features (e.g., if a patient is near a high-risk location such as a familiar bar, a GPS-initiated alert asks the patient if s/he really wants to be there)
ORION	The Overdose RIsk InfOrmatioN project was set up to develop and pilot an e-health psychoeducational tool that provides information about the risks of having a drug overdose
D-ARIANNA	The Digital-Alcohol RIsk Alertness Notifying Network for Adolescents and young adults was set up to develop and pilot an e-health psychoeducational tool that provide information to adolescents and young adults about the risks of binge drinking
Steering Clear	Steering Clear of Driving After Drinking is a tailored e-health intervention that aims to reduce repeat offending by first-time convicted drink driving offenders via an online program
JITAI	The Just-In-Time Adaptive Intervention framework could be used to design a mobile app that carries out in-the-moment monitoring of triggers for lapsing, and delivers personalized coping strategies to prevent lapses from occurring
reSET	reSET is a digital therapeutic system designed to be used as an adjuvant to standard outpatient therapy for treating SUDs. It combines patient-facing interventions and assessments via a mobile device, with clinician-facing dashboards and data analytics on the back end

### Machine Learning

Machine learning is the subfield of artificial intelligence that gives “computers the ability to learn without being explicitly programmed” [Arthur Samuel, 1959 (30)]. It uses a different kind of classification process: *Supervised* classification seeks to automatically identify rules from databases constituted of “examples” (classically, these are cases that have already been validated, such as a well-established diagnosis), while with *unsupervised* classification, the collected data are not labeled, and the objective of the software is to group them into clusters so that the closest and most similar ones are placed in the same cluster, whereas those that are further apart are placed in separate clusters. This method makes it possible to find new structures. The coupling of machine learning with complementary examinations (MRI, EEG) reveals patterns that allow patients to be divided into different groups, which could be useful for screening or for describing groups with a particular phenotype (e.g., patients at risk of relapse). These techniques will make it possible in the near future to improve addictologists’ predictive skills, as is already the case for the prediction of psychotic transition in patients in an at-risk mental state (31) and in the field of mood disorders (32, 33). In addictive medicine, one study (34) has already shown that the risk of alcohol relapse can be predicted with 77% accuracy by analyzing clinical data (e.g., demographics, alcohol use, dependence severity, craving, health-related quality of life, and psychological measures at baseline), while the probability of treatment success can be predicted with an AUC between 0.793 and 0.820 (35) using clinical data (10 patient characteristics, 3 treatment characteristics, principal source of referral, summary of type of problematic substance and mental health problem). Chih et al. (36) used a Bayesian network model to predict relapse, based on responses to 2934 A-CHESS weekly surveys provided by 152 alcohol-dependent individuals who had recently completed residential treatment. It showed good predictability, with an AUC between 0.829 (cross-validation) and 0.912 (external validation). Mumtaz et al. (37) developed a machine learning method that utilized resting-state EEG-derived features as input data to distinguish patients with alcohol use disorder from healthy controls and to perform automatic screening. Results showed that interhemispheric coherences between brain regions differed significantly between the study groups, with high classification efficiency (accuracy = 80.8, sensitivity = 82.5, specificity = 80; *F* = 0.78). The authors concluded that EEG data can be used as objective markers for screening patients with alcohol use disorder.

In the field of addictology research, these techniques are now being used to identify behavioral biomarkers predictive of the use of substances such as cocaine (38) or heroin (39).

### Computerized Adaptive Testing

Computerized Adaptive Testing (CAT) (40) has been developed to *mimic* clinicians. It uses a limited form of artificial intelligence to automatically adapt questionnaire items to the answers provided by the patient to previous items, using a large database of possible answers/questions. More specifically, after the first general questions, an algorithm adapts the subsequent items according to the patient’s initial answers. Complementary questions enhance the accuracy of the evaluation. The advantages of this type of testing are therefore an improvement in performance and a reduction in test duration, which is important, as the difficulties reported by patients mostly concern the amount of time spent completing the scales and the repetitiveness of the questions. This method, first developed by Fliege in 2005 and replicated by Gibbons (41), has been validated in depression disorders (42) with the CAT-DI. In the field of addictions, Pilkonis (43) demonstrated the validity of this method using the Patient-Reported Outcomes Measurement Information System (PROMIS), which includes five item banks for alcohol use. PROMIS CATs has been shown to be efficient and makes it feasible to use a comprehensive health status profile in a substance use treatment setting, providing important prognostic information regarding abstinence and drinking severity. Versions of this tool can also be used to rapidly explore common comorbidities with SUDs, such as the CAT-SS for suicidal behaviors (44) or the CAT-ANX for anxiety (45). Some related tests are now being developed using virtual *avatars* of psychiatrists who can converse directly with patients, known as *embodied conversational agents* (46).

### Wearable Devices and Digital Phenotyping

John Torous and Lisa Gualtieri recently recalled the potential worth of connected objects in the field of mental health (47), as many devices now include multiple sensors such as accelerometers, heart rate sensors, sleep trackers, skin conductance sensors, and light sensors. The potential to gather real-time physiological data from fitness trackers, with the addition of symptom surveys from smartwatches, is attractive, and there is increasing interest in using real-time patient data as biomarkers of illness (48).

John Torous et al. have developed the concept of the *digital signature* or *digital phenotype* of pathology. These terms refer to the capture by computerized measurement tools of specific characteristics of psychiatric disorders (49, 50). Some behaviors or symptoms can be objectified and quantified by computer tools, constituting an *e-semiotics*. Sensor miniaturization and the ubiquitous use of smartphones make it possible to collect a large amount of data that psychiatrists had never been able to access before. Models based on these new *semiological signs* are beginning to emerge (51, 52). This collection uses *passive* data, for which no intervention is necessary, as they are collected in *background tasks*, sometimes without the patient realizing it. The objective is to reduce the influence of the observer as much as possible. This detection may involve both a mobile phone and its onboard sensors (GPS, accelerometer, verbal flow detector, etc.) or else connected wearable objects that enable biometric monitoring in real time. For example, it is now possible to use heart rate variability (HRV) to distinguish an alcohol-dependent patient from a nondependent chronic alcohol user. Defined as the degree of fluctuation in the interval between two cardiac contractions, HRV is dependent on the autonomic nervous system and is markedly decreased in dependent patients (53). The links between HRV and addictive disorders, alcohol dependence in particular, are now quite well known (54). It is an interesting biomarker, albeit not very specific, as changes in HRV are also encountered in mood disorders and posttraumatic stress disorder (PTSD) (55). In addictology, there has not yet been any research on specific digital phenotypes for different types of substance use, but more and more researchers are interested in coupling EMA with GPS (56, 57) or biosensor data. Activity-space analysis, which examines motion in different contexts, and EMA, which captures microlevel contextual changes as individuals move through their day can, for instance, improve understanding of drinking contexts in alcohol studies. Better identification of drug-using contexts can trigger the implementation of targeted interventions to prevent acting out.

Wearable biosensors have been developed to:

- Study physiological change during opioid use (decrease in locomotion and increase in skin temperature are consistently detected) (58);
- Monitor real-time drug use (59) or alcohol consumption (60), possibly through the detection of a metabolite (ethyl glucuronide) in human sweat (61);
- Monitor and study autonomic nervous system activity via electrodermal activity, 3-axis acceleration, ECG and temperature, in order to detect arousal events and automatically send therapeutic and empathetic messages to the patient using CBT (62).

These sensors can be coupled with biofeedback systems (cf. 2.6).

### EMA and EMI

Classic data collection relied on a conventional interview format where the psychiatrist observes, questions, and evaluates the patient in order to form an opinion about that patient’s diagnosis, consumption, and the consequences of that consumption. EMA is the evaluation of symptoms day to day, in the patient’s usual environment, free from recall biases as the patient self-assesses *right then, not later; right there, not elsewhere* (10, 11). This new method using active data (supplied by the patient) profoundly modifies the assessment procedure by introducing a computerized third party between doctor and patient. The use of dedicated smartphone apps allows patients to keep an accurate diary of their symptoms, behaviors, or consumption. Studies have shown that EMA apps are just as reliable as the scales usually used for psychiatric disorders (49, 63), with excellent acceptability, and possibly even a preference for this medium. Several studies [for a review, see in Ref. (13, 64)] have allowed SUDs to be assessed in real time:

- Alcohol use (12);
- Relationship between alcohol use and PTSD symptom intensity (65);
- Relationship between alcohol use and mood (66, 67);
- Real-time illicit drug use (68, 69);
- Opioid craving (70);
- The context or state of mind in which a patient consumes (71);
- Effects of stress on relapse (72);
- Effects of Topiramate on alcoholic craving (73);
- Physical, interpersonal, or legal consequences of SUD (74).

Moreover, this repeated evaluation of symptoms over time may in itself have a therapeutic effect. A study conducted among patients with bipolar disorder (75) found that it potentially limits manic or hypomanic episodes, and we can assume that repeated assessment of addictive behaviors also has a therapeutic effect. The feeling of intrusion generated by self-report questionnaires is seldom reported in these studies, indicating good acceptability (76). Compliance is variable and depends on the patient’s type of consumption (77). For example, cannabis users are the least compliant, and a study is currently ongoing (78).

Smartphone-based intervention can take the form of either a virtual coach (79) applying CBT therapies or else a Screening, Brief Intervention, and Referral to Treatment (SBIRT) program (80, 81) that evaluates the patient quickly and offers appropriate care. Mobile phones afford the possibility of undertaking EMIs, that is, targeted, one-off interventions if a patient risks relapse or consumption. These targeted interventions can take many forms (12–14):

- SMS messages;
- Psychoeducation information;
- Realtime coping strategies;
- Motivational messages;
- Behavioral change promotion (82).

These tools construct a genuinely protective network (22) around the patient, who can benefit at any time from a healthcare platform offering information on possible care (including emergency care).

### Biofeedback and Neurofeedback

Developed in the 1970s (16), biofeedback is a painless, noninvasive procedure that consists in capturing biometric data (EEG, ECG, EMG, skin conductance, temperature) and feeding them back to the patient in real time. The objective of neurofeedback, the name given to biofeedback measuring brain activity (by EEG or real-time fMRI), is to model the patient’s brain activity in real time as an image (video game type) or a sound. Based on CBT techniques and relaxation, patients gradually learn (through positive reinforcement) to promote brain activity corresponding to the therapeutic target. When activity in a desirable frequency band increases, the symbol modeling the brain activity changes (e.g., the video game moves faster), whereas when activity in an unfavorable band increases, the symbol changes in the opposite direction (e.g., the video game slows down). Patients gradually *learn* the newbrain wave, taking a wave corresponding to what is observed in healthy individuals as their model. Biofeedback research is currently focused on a variety of clinical issues (83), and several studies have examined the treatment of addictions by neurofeedback (84–87), especially for opiates and alcohol. One therapeutic hypothesis is that striatal cue reactivity to alcohol stimuli is reduced after neurofeedback training (88). A clinical study is in progress to compare neurofeedback training for alcohol dependence with classic treatment (89).

### Virtual Reality

Until recently, virtual reality was limited by its cost and by the quality of the multimedia content. There has been a recent democratization of these systems (PS4 VR, Occulus Rift, etc.) concomitant with the video game industry’s growing interest in this technology. Decreasing costs and increasing power are making it useful for performing neuropsychological (cognition, emotions, and behavior) assessments in real-time (90). Both the environment and the perceptual stimuli can be manipulated to trigger pathological behaviors or sensations (e.g., craving) as well as to evaluate behavioral responses to a given situation that can elicit distress, allowing patients to learn how to cope with their problems better. Many studies are therefore underway, with a focus on environmental trigger disorders (anxiety disorders in particular), but also on depression. Eichenberg and Wolters (91) conducted a descriptive review of virtual reality studies prior to 2012 that was subsequently complemented with studies up to 2015 by Valmaggia et al. (92). These reviews showed that the most commonly treated disorders are anxiety disorders, eating disorders, schizophrenia (distress associated with hallucinations or delusions), and PTSD. Virtual reality therapy has good acceptability (93) and has been shown to be more effective in comparisons with patients receiving standard treatment or wait list control groups. Its results are similar to those of conventional CBT or *in vivo* exposure. Neither review contained data on SUDs, but several recent studies have been conducted in alcohol addiction care, online gaming addiction (94, 95), and opioid use (96). Concerning alcohol dependence treatment, a study featuring a combination of relaxation, presentation of a high-risk situation, and presentation of an aversive situation (97) highlighted a neurobiological imbalance (high sensitivity to stimuli) in the limbic system in individuals with alcohol dependence. This protocol could potentially have a regulating effect on limbic circuits. Several studies (98–100) examining craving triggers and control have found that patients with alcohol dependency report extremely high levels of craving immediately upon exposure to a virtual environment with alcohol cues, regardless of social pressure, social drinkers’ alcohol use is strongly influenced by their social environment. The use of virtual reality in the treatment of SUDs therefore involves exposure to the stimulus that induces craving, either via situational cues (social environment) or via the implementation of alcohol-based cues, allowing patients’ coping skills to be tested in real time. Finally, as in surgery, virtual reality can be used for training purposes to enhance screening or intervention methods for caregivers who may be confronted with SUDs (101).

## Discussion

There is a constantly growing body of knowledge in addictive medicine, and it is becoming increasingly complicated to handle all these data on a daily basis. In addition, more validated tools are needed to optimize the management the complexities of addiction. In the present overview, we discuss the major concepts related to new technologies that may well provide solutions in the field of addiction evaluation, diagnosis, or therapy within the near future. E-psychiatry is already booming, and some even talk about a *digital mental health revolution* (102). The acceptability of these technologies must therefore be assessed at different levels. It is generally based on several major criteria: usability (device’s flexibility and ease of learning), utility (technology’s contribution), satisfaction and reliability (including accuracy, effectiveness and efficiency). Cost, although fundamental, is a secondary consideration. Finally, the concept of risk impinges on acceptability and constitutes an important dimension of medical reasoning. It must therefore be taken into account when these technologies are being assessed (impact of false positives or false negatives, ethical issues).

In the present overview, we showed that these tools could prove extremely useful. The improved communication between healthcare providers via web-based, computer-based, or smartphone-based interventions can facilitate the management of a range of psychiatric or behavioral disorders. Some software (CAT) can support (and sometimes replace) clinicians in screening and monitoring. The recent development of digital phenotyping, where a computer collates the clinical characteristics of a mental state (sometimes with greater accuracy than a clinician), and the possibility of doing so remotely, will undoubtedly modify current practice. Most authors advocate the use of passive rather than active data in the context of disorders for which there is some anosognosia (e.g., bipolar disorder, SUDs), as this type of automatically generated data makes it possible to limit biases and the feeling of intrusion that EMA and self-report questionnaires can generate (especially if they are to be filled out regularly or appear in pop-ups).

Confidence in e-health among patients with addictions and healthcare professionals is a major issue (103). Studies have highlighted good acceptability and patient compliance (except for patients with a cannabis addiction), as there is no feeling of being *observed*. Using a smartphone seems less stigmatizing than using a specific device (e.g., connected bracelet). Machine learning is revolutionizing fundamental research by allowing for better classification of patients, based not only on clinical data, but also on biological or neuroimaging-derived data. It is becoming reasonable to talk about genuinely complementary examinations in behavioral studies. Finally, these new technologies are enabling the development of new therapies, including biofeedback and virtual reality, which focus on craving control and the learning of coping skills.

Although there is mounting evidence that e-addictology offers new opportunities for treatment, there is a lack of randomized controlled trials. Available studies have several limitations, including small sample sizes, heterogeneous study samples, only short-term follow up, and difficulty in determining whether the treatment effects were restricted to the studied addiction or could be generalized to other types of addiction. For example, smartphone apps are steadily increasing in number, but most are of a commercial nature and not truly efficient for patients and practitioners (104). There is also a gap between patients’ assessments and evidence-based medicine: a *like* is not a statistical difference. Users may thus be exposed to dangerous recommendations, and in any case, the overall agreement between guidelines and their content is often very low (105). Another concern is liability in the case of the malfunction of products, sensors, software or in security (hacking of data for example). There are also potential errors in false-positives and false-negatives. Data must therefore be objectively analyzed to avoid the use of tools with low validity and reliability. Physicians obviously need to be involved in the development and evaluation of these tools.

There is also a dearth of information regarding the cost-effectiveness of e-health tools and services (106). The lack of reimbursement schemes is equally problematic. Drug users live in more precarious conditions than the general population in terms of housing, social protection, and resources. The use of these new technologies may therefore be out of the reach of people with low incomes and/or limited computer literacy.

Lastly, the development of connected health technologies raises many ethical issues, the most important probably being the protection and ownership of personal and health data. The issues of confidentiality and transparency regarding data use have yet to be resolved. Very few patients are currently willing for their data to be shared with private companies (107).

These changes call for a shift in thinking and in ways of doing things, and we can still ask what technological help addictologists would welcome and for which tasks? Can they bear comparison with certain technologies that sometimes seem to exhibit greater predictive accuracy? Or can their role as physicians be better asserted through these devices? A study (108) that used a scenario-based methodology (evaluating the predictive value of medical imaging and passive vs. active data collection) to explore the acceptability of these new technologies among 515 French psychiatrists (including addictologists) revealed considerable disparity in acceptability, depending on the psychiatrists’ profiles. Addictologists (*n* = 34), were among those who best accepted these new technologies, deeming that they could usefully support the therapeutic relationship, and who did not feel at all threatened by these devices.

## Conclusion

Active data obtained from EMA can provide a means of assessing addictive behaviors (intrusion, sleep, etc.) and gaining an idea of their severity. The advantages of passive data gathering through smartphones, biosensors or connected objects, artificial intelligence, and the remote monitoring of patients with psychiatric pathologies have yet to be defined, and the question of data security will soon become central. In order to prevent data from being used for nonmedical purposes, we believe it is essential that physicians take up this issue and make recommendations on the subject. Important ethical considerations are hampering the acceptance of these technologies. If they are to be used, these new tools must therefore be explained and adapted to physician and patient profiles, all the while taking account of the risks inherent to their use (data piracy, false positives, etc.). Patients, caregivers, and other health professionals need to be involved in the design and evaluation of these new tools.

## Author Contributions

FF and AB: both authors contributed equally to this work, screening and abstract analysis, writing, conceptual framework. SM: supervised development of work, helped in data interpretation and manuscript evaluation. LK: helped to evaluate and edit the manuscript.

## Conflict of Interest Statement

FF, AB, and SM have no conflict of interest. LK receives consulting fees from BMS Otsuka, Lundbeck, Gillead, Shering Plough, Eutherapie, Merck/Serono, Astra Zeneca, Janssen-Cilag, Bouchara Recordati Pharmaceuticals.
